# Diabetes mellitus type 2 and other chronic non-communicable diseases in the central region, Saudi Arabia (riyadh cohort 2): a decade of an epidemic

**DOI:** 10.1186/1741-7015-9-76

**Published:** 2011-06-20

**Authors:** Nasser M Al-Daghri, Omar S Al-Attas, Majed S Alokail, Khalid M Alkharfy, Mansour Yousef, Shaun Louie Sabico, George P Chrousos

**Affiliations:** 1 Biomarkers Research Program, Biochemistry Department, College of Science, King Saud University, Riyadh 11451, Kingdom of Saudi Arabia; 2 Center of Excellence in Biotechnology Research, King Saud University, Riyadh, 11451, Kingdom of Saudi Arabia; 3 Clinical Pharmacy Department, College of Pharmacy, King Saud University, Riyadh 11451, Kingdom of Saudi Arabia; 4 Health Affairs for Riyadh Region, Ministry of Health, 11176 Riyadh, Kingdom of Saudi Arabia; 5 Division of Endocrinology, Metabolism & Diabetes, University of Athens Medical School, Children's Hospital Aghia Sophia, Athens 115 27, Greece

## Abstract

**Background:**

Follow-up epidemiologic studies are needed to assess trends and patterns of disease spread. No follow-up epidemiologic study has been done in the Kingdom of Saudi Arabia to assess the current prevalence of major chronic, noncommunicable diseases, specifically in the urban region, where modifiable risk factors remain rampant. This study aims to fill this gap.

**Methods:**

A total of 9,149 adult Saudis ages seven to eighty years (5,357 males (58.6%) and 3,792 females (41.4%)) were randomly selected from the Riyadh Cohort Study for inclusion. Diagnosis of type 2 diabetes mellitus (DMT2) and obesity were based on the World Health Organization definitions. Diagnoses of hypertension and coronary artery disease (CAD) were based on the Seventh Joint National Committee on Prevention, Detection, Evaluation, and Treatment of High Blood Pressure and American Heart Association criteria, respectively.

**Results:**

The overall crude prevalence of DMT2 was 23.1% (95% confidence interval (95% CI) 20.47 to 22.15). The age-adjusted prevalence of DMT2 was 31.6%. DMT2 prevalence was significantly higher in males, with an overall age-adjusted prevalence of 34.7% (95% CI 32.6 to 35.4), than in females, who had an overall age-adjusted prevalence of 28.6% (95% CI 26.7 to 29.3) (*P *< 0.001). The overall crude prevalence of obesity was 31.1% (95% CI 30.1 to 32.0). The age-adjusted prevalence of obesity was 40.0%. The prevalence of obesity was higher in females, with an overall prevalence of 36.5% (95% CI 35.1 to 37.83), than in males (25.1% (95% CI 23.7 to 26.3)) (*P *< 0.001). The age-adjusted prevalence of hypertension and CAD were 32.6% (95% CI 31.7 to 33.6) and 6.9% (95% CI 6.4 to 7.4), respectively.

**Conclusion:**

Comparisons of our findings with earlier data show that the prevalence of DMT2, hypertension and CAD in Riyadh, Saudi Arabia, has alarmingly worsened. Aggressive promotion of public awareness, continued screening and early intervention are pivotal to boosting a positive response.

## Background

If the prevalence of diabetes mellitus type 2 (DMT2) continues to increase at the current rate, the global burden of this disease will swell between 2000 to 2030 from 171 million to 366 million patients [1]. Furthermore, healthcare expenditures on DMT2 alone will skyrocket from US$376 billion in 2010 to US$490 billion in 2030 [2]. The Middle East region has not been spared from this scourge and currently is among those worst-hit [1]. This global epidemic, shared by both industrialized and developing countries, has stimulated increased public awareness of the disease, the identification of risk factors and the knowledge that DMT2 can be delayed and, even better, prevented [3-5]. Recognition of the importance of glycemic control in the prevention of the complications and morbidity of DMT2 has led to worldwide campaigns for modifications in lifestyle and an intensive search for better antidiabetes medications [6-8].

In the Kingdom of Saudi Arabia (KSA), the rise in the prevalence of DMT2 started to gain attention years after rapid industrialization took place in the country [9]. Studies done since the late 1980s have shown an increasing trend among adult Saudis [10-12], the last of which, conducted in a large cohort of patients assembled from 1995 to 2000, revealed that one of five adult Saudis had DMT2 [13]. The same cohort showed an alarming prevalence of obesity at 40.0%, hypertension at 30% and coronary artery disease (CAD) at 6.2% [14-16]. A decade passed, and a follow-up epidemiologic study was designed to assess the current status of the population and whether the efforts of the Ministry of Health and the healthcare community have borne fruit.

## Methods

The patients were part of the capital-wide Biomarker Screening in Riyadh (BSR), an ongoing collaborative effort between the Biomarkers Research Program (BRP) of King Saud University and the Ministry of Health in Riyadh, KSA (RIYADH Cohort). In brief, BSR was launched to identify and employ novel biomarkers of chronic noncommunicable diseases, including diabetes mellitus (DM), cardiovascular diseases, hypertension and obesity, among consenting Saudis. Ethical approval was obtained from the Ethics Committee of the College of Science Research Center of King Saud University, Riyadh, KSA.

Patients' information was taken from the existing database of more than 17,000 individuals. In this cross-sectional, observational study, a total of 9,149 Saudis (5,357 males (58.6%) and 3,792 females (41.4%)) ages seven to eighty years old were included. Patients were recruited randomly from their homes using the cluster sampling strategy. They were invited to visit the nearest primary healthcare center (PHCC). These centers span the entire Riyadh region. The population of each PHCC was taken as a cluster, and the allocations of the required numbers of patients were proportional to the populations served by the PHCCs. No expatriates were included in the conduct of this study. Each participating patient filled in a general questionnaire containing demographic, past and present medical history, as well as diet information from the food frequency questionnaire. This questionnaire was developed, pretested and validated in a pilot study. Informed written consent was obtained from each patient prior to inclusion.

### Anthropometrics

Anthropometry included height (rounded off to the nearest 0.5 cm) and weight (rounded off to the nearest 0.1 kg), which were measured using an appropriate international standard scale (Digital Person Scale; ADAM Equipment, Milford, CT, USA), as well as waist and hip circumference in centimeters, which were measured using a standard tape measure. Mean systolic and diastolic blood pressure readings (in mmHg; average of two readings) were taken using appropriate cuffs.

### Biochemical measurements

Consenting adults were invited to their respective PHCCs after a 10-hour overnight fast. Blood was drawn, centrifuged and processed on the same day. Both whole blood and serum were placed in plain polystyrene tubes. Serum was delivered to BRP for storage at -20°C. Fasting serum glucose was quantified using routine laboratory analysis (Konelab, Espoo, Finland). This biochemical analyzer was calibrated routinely prior to the analysis of all serum samples using quality control samples provided by the manufacturer (ThermoFisher Scientific, Espoo, Finland). The glucose-measuring method employed a glucose oxidase and modified Trinder color reaction catalyzed by the enzyme peroxidase.

### Diagnosis of chronic noncommunicable diseases

The World Health Organization (WHO)-proposed cutoffs for DMT2 and impaired fasting glucose (IFG) were used. DMT2 was associated with a fasting plasma glucose (FPG) level ≥7.0 mmol/L (126 mg/dL), IFG with FPG level between 6.1 and 6.9 mmol/L (110 to 125 mg/dL) and normal with a FPG level < 6.1 mmol/L (110 mg/dL). All patients whose glucose levels exceeded the cutoffs were referred back to their respective PHCC physician for further tests and classification of DM. Screening for adult hypertension was based on the recommendations of the Seventh Joint National Committee on Prevention, Detection, Evaluation, and Treatment of High Blood Pressure [17]. Body Mass Index (BMI) was calculated as body weight in kilograms divided by height in square meters. Overweight was defined as a BMI of 25 to 29.9 kg/m^2^, and obesity was defined as a BMI ≥30. For patients ages 7 to 17 years, the cutoffs proposed by Cole and colleagues [18] were used for the definition of overweight and obesity. Diagnosis of hypertension in children was based on percentile [19]. CAD patients were known cases based on a medical history of angiography and intake of antiarrhythmic drugs.

### Data analysis

Statistical analyses were carried out using SPSS 16.0 for Windows software (SPSS Inc., Chicago, IL, USA). The prevalence data for each of the various patient groups are shown with 95% confidence intervals. Prevalence was also calculated for gender and various age strata.

## Results

The overall crude prevalence of DMT2 was 23.1% (95% confidence interval (95% CI) 20.47 to 22.15). The age-adjusted prevalence of DMT2 was 31.6%. The overall prevalence of IFG, on the other hand, was 9.0% (95% CI 8.37 to 9.53), with an age-adjusted prevalence of 10.2%. The prevalence of DMT2 was significantly higher in males, with an overall age-adjusted prevalence of 34.7% (95% CI 32.6 to 35.4), than in females (28.6% (95% CI 26.7 to 29.3)) (*P *< 0.001). The overall crude prevalence of obesity was 31.1% (95% CI 30.1 to 32.0). The age-adjusted prevalence of obesity was 40.0%. The overall prevalence of overweight, on the other hand, was 26.3% (95% CI 25.3 to 27.2), with an age-adjusted prevalence of 30.8%. The prevalence of obesity was significantly higher in females, with an overall prevalence of 36.5% (95% CI 35.1 to 37.83), than in males (25.1% (95% CI 23.7 to 26.3)) (*P *< 0.001). The overall prevalence of CAD was 4.2% (95% CI 3.9 to 4.7), with an age-adjusted prevalence of 6.9% (95% CI 6.4 to 7.4). The overall prevalence of hypertension was 25.7% (95% CI 24.8 to 26.6), with an age-adjusted prevalence of 32.6% (95% CI 31.7 to 33.6).

**Figure 1:**
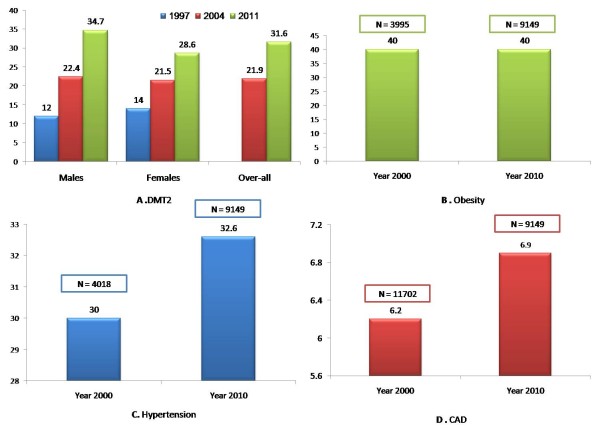
**Trends in the prevalence of chronic noncommunicable diseases in the Kingdom of Saudi Arabia from 2000 to 2010**. (**A**) Age-adjusted prevalence of type 2 diabetes mellitus in Saudi Arabia according to gender (1997 [36], 2004 [7], 2011 (present study)). (**B**) Coronary artery disease. (**C**) Hypertension. (**D**) Obesity. Previous estimates shown are from the central region (Riyadh) and do not include other regions.

Tables 1 and 2 show the overall prevalence of DMT2 and the rest of the nonchronic diseases, as well as the gender-stratified prevalence according to age. Figure 1 shows the increasing trend of DMT2 from 1997 to 2011, as well as data for other chronic noncommunicable diseases in comparison to previous estimates.

**Table 1:** Prevalence of chronic noncommunicable diseases according to age group^a^

Age group, yr	Patients, *n*	DMT1	DMT2	GDM	IGT	Overweight	Obese	HPN	CHD
7 to 17	2,519	0.4 (0.15 to 0.65)	4.5 (3.72 to 5.34)		5.6 (4.7 to 6.5)	16.6 (15.18 to 18.08)	12.54 (11.25 to 13.8)		
18 to 45	3,954	1.3 (0.94 to 1.64)	12.2 (11.14 to 13.18)	1.39 (1.03 to 1.75)	9.6 (8.64 to 10.48)	27.9 (26.5 to 29.35)	33.4 (31.9 to 34.8)	14.0 (12.9 to 15.1)	1.5 (1.2 to 1.9)
46 to 60	1,758	3.13 (2.32 to 3.94)	46.7 (44.37 to 49.03)	1.5 (0.92 to 2.04)	12.1 (10.54 to 13.58)	31.8 (29.6 to 33.9)	50.5 (48.2 to 52.8)	40.7 (38.4 to 43.0)	10.4 (8.9 to 11.8)
61 to 80	918	4.79 (3.41 to 6.17)	58.2 (54.9 to 61.4)		9.6 (7.7 to 11.5)	35.1 (31.9 to 38.2)	34.5 (31.4 to 37.6)	58.2 (55.0 to 61.4)	14.5 (12.3 to 16.8)

^a^DMT1, type 1 diabetes mellitus; DMT2, type 2 diabetes mellitus; GDM, gestational diabetes mellitus; IGT, impaired glucose tolerance; HPN, hypertension; CHD, coronary heart disease. Data are presented as percentages (95% confidence interval).

**Table 2:** Prevalence of chronic noncommunicable diseases according to gender^a^

Gender	Age group, yr	Patients, *n*	DMT1	DMT2	GDM	IGT	Overweight	Obese	HPN	CHD
Men	7 to 17	1,376	0.4 (0.1 to 0.8)	4.6 (3.5 to 5.7)		5.6 (4.4 to 6.8)	15.2 (13.3 to 17.1)	12.0 (10.3 to 13.7)	1.3 (0.71 to 1.9)	0
	18 to 45	1,581	1.83 (1.2 to 2.5)	15.8 (14.0 to 17.5)		10.9 (9.4 to 12.4)	29.4 (27.2 to 31.7)	28.8 (26.6 to 31.1)	16.8 (14.9 to 18.6)	2.3 (1.6 to 3.1)
	46 to 60	823	2.94 (1.8 to 4.1)	49.9 (46.5 to 53.4)		9.9 (7.8 to 11.9)	39.0 (35.7 to 42.4)	37.6 (34.3 to 40.9)	41.6 (38.2 to 45.0)	11.2 (9.1 to 13.4)
	61 to 80	586	4.1 (2.5 to 5.7)	61.0 (57.0 to 64.9)		7.9 (5.7 to 10.1)	37.6 (33.7 to 41.6)	27.8 (24.2 to 31.4)	57.2 (53.2 to 61.2)	16.2 (13.1 to 19.2)
Women	7 to 17	1,143	0.4 (0.1 to 1.0)	4.3 (3.1 to 5.5)		5.5 (4.2 to 6.9)	18.5 (16.3 to 20.8)	12.9 (10.9 to 14.8)	1.4 (0.72 to 2.1)	0
	18 to 45	2,373	0.9 (0.6 to 1.3)	9.6 (8.4 to 10.8)	1.39 (1.03 to 1.75)	8.6 (7.5 to 9.8)	26.9 (25.1 to 28.7)	36.4 (34.5 to 38.4)	12.2 (10.8 to 13.5)	1.03 (0.62 to 1.4)
	46 to 60	935	3.23 (2.1 to 4.4)	44.1 (40.8 to 47.3)	1.5 (0.92 to 2.04)	13.9 (11.6 to 16.1)	25.4 (22.6 to 28.2)	61.9 (58.7 to 64.9)	39.9 (36.7 to 43.0)	9.7 (7.8 to 11.6)
	61 to 80	332	6.08 (3.5 to 8.6)	53.2 (47.8 to 58.6)		12.4 (8.9 to 16.0)	30.7 (25.7 to 35.7)	46.5 (41.1 to 51.9)	61.1 (55.8 to 66.4)	12.1 (8.6 to 15.7)

^a^DMT1, type 1 diabetes mellitus; DMT2, type 2 diabetes mellitus; GDM, gestational diabetes mellitus; IGT, impaired glucose tolerance; HPN, hypertension; CHD, coronary heart disease. Data are presented as percentages (95% confidence interval).

## Discussion

The last overall crude prevalence of DMT2 in the KSA, as well as the central region in particular (Riyadh), was 23.7%, with an age-adjusted prevalence of 21.9% [7]. In the present study, the crude prevalence was 21.3%, with a calculated age-adjusted prevalence of 31.6%. The slight decrease in crude prevalence in the current study is negligible, considering the age range of the previous study, which was ages 30 to 70 years compared to ages 7 to 80 years in this study. Of note, in the previous report, 3,883 patients from the central region were studied, as opposed to the 9,149 patients included in this study. Interestingly, the age-adjusted prevalence of obesity in the central region was previously 40.0%, the same prevalence observed in the current study. These findings suggest that obesity is a major contributor to DMT2, but not the only one, and, in this instance, cannot explain the observed rise in the prevalence of the disease in the KSA.

It is apparent that the prevalence of DMT2 in the region has worsened, and the war against DMT2 in the kingdom is still far from over. Sadly, our results are beyond the predicted estimates of Shaw and colleagues [20], who stated that the biggest increase in the number of patients with DMT2 will be in developing nations. Several reasons may account for the higher prevalence of DMT2 in the KSA. The prevalence of conventional risk factors for DMT2, such as the full metabolic syndrome (MetS) and its individual manifestations, are still alarming among adult Saudis, 37% of whom have the full MetS [21], while just recently partial Metabolic syndrome (MetS) and isolated MetS manifestations were documented to be extremely high even among Saudi children [22]. In both cases, dyslipidemia accounted for almost 90% of the patient population [21,22].

The emergence of novel risk factors for insulin resistance in this population has also come into play and includes, but is not limited to, vitamin D deficiency [23,24], smoking cessation [25] and deficient sleep [26]. Treatment-wise, diabetes care in the primary care setting remains far from desirable [27], and, while limited studies have been done to assess public health awareness of DMT2 in the KSA, one recent study revealed poor knowledge of DMT2 risk factors and preventive measures among Saudi patients in the eastern region [28]. In addition, the general population still has an extremely high prevalence of physical inactivity, which is a major risk factor for all the diseases included in the study, including hypertension [29,30]. Last, granted that DMT2 and other chronic diseases are polygenic disorders, the high consanguinity marriages among the Saudi population might be a contributing factor [31]. Any combination of the above risk factors, as well as an increase in the exposure to stress, might explain why the prevalence of chronic diseases in Saudi adults has increased even in the absence of a change in the prevalence of obesity [32].

In our study, there was an apparent gender difference in the prevalence of DMT2, with the prevalence in men higher than that in women. Such a difference was documented in a similar screening program done in the central region in the 1990s [33]. While it is known that men generally have a shorter life expectancy than women, several studies have highlighted lifestyle risk factors that are more frequent among Saudi males, including tobacco smoking [34], obesity and eating habits [35] and, just recently, vitamin D deficiency [36]. These factors cumulatively, but not completely, explain why there are more Saudi men than women with DMT2 and other chronic noncommunicable diseases. Furthermore, perhaps, the KSA and the Arabian Peninsula in general maintain a highly patriarchal society, which may place men at higher levels of chronic psychological stress than women, which in turn may contribute to metabolic and inflammatory stress over time, leading to several cell aging mechanisms and ultimately to chronic noncommunicable diseases such as DMT2 and CAD [37,38].

With the exception of gestational diabetes mellitus (GDM) and CAD, the burden of otherwise adult-onset, chronic, noncommunicable diseases mentioned in this study is most striking and alarming in the pediatric cohort. This finding further expands and complicates the already challenging burden of these diseases. Aside from the environmental factors already mentioned, we recently demonstrated, in families from the same cohort, that multiple metabolic parameters, including circulating levels of key adipocytokines such as leptin and adiponectin, are significantly transmitted from parents to their offspring, with this phenomenon manifesting as early as the preteen years [39]. This finding, compounded by a metabolically adverse gestational and postnatal environment, which is prevalent in the KSA [40], adds to the susceptibility of the already genetically predisposed individual to a lifetime of insulin resistance and related morbidities.

We acknowledge several limitations of the present study. The prevalence of CAD was based only on the number of known cases, so the true prevalence of CAD in the cohort is expected to be higher. Furthermore, while current estimates may not necessarily reflect the true prevalence at a national level, since patients were recruited only in the central region, the findings are, on their own, strongly indicative of the need for a more aggressive approach to battle this epidemic.

## Conclusions

In conclusion, this follow-up epidemiologic study shows that the prevalence of DMT2 in the KSA, specifically in its central region, has increased by a whopping 10.0% in just a decade, while it shows no improvement in the age-adjusted prevalence of obesity and a modest increase in the prevalence of hypertension and CAD. Translating these findings to aggressive health policies at the grassroots level and strict implementation of diabetes care is necessary to efficiently restrain the disease. Emerging risk factors for DMT2 specific to the Saudi population should also be addressed and further explored.

## Competing interests

The authors declare that they have no competing interests.

## Authors' contributions

NMA and OSA conceived of the study. MSA, KMA and MY carried out data acquisition and interpretation. SLS and GPC analyzed the data and prepared the manuscript. All authors provided intellectual contributions to the manuscript and read and approved the final version.

## Pre-publication history

The pre-publication history for this paper can be accessed here:

http://www.biomedcentral.com/1741-7015/9/76/prepub

## References

[B1] Wild S Rogli G Green A Sicree R King H Global prevalence of diabetes: estimates for the year 2000 and projections for 2030 Diabetes Care 2004 27 1047 1053 10.2337/diacare.27.5.1047 15111519

[B2] Zhang P Zhang X Brown J Vistisen D Sicree R Shaw J Nichols G Global healthcare expenditure on diabetes for 2010 and 2030 Diabetes Res Clin Pract 2010 87 293 301 10.1016/j.diabres.2010.01.026 20171754

[B3] Cole A Nathan DM Spavaria-Porter E Copeland P Turchin A Brunt M Zusman R Barrett JA Wexler D Case E McMahon GT Mort E An algorithm for the care of type 2 diabetes Crit Pathw Cardiol 2009 8 156 165 19952550 10.1097/HPC.0b013e3181c017e2

[B4] Rodbard HW Jellinger PS Davidson JA Einhorn D Garber AJ Grunberger G Handelsman Y Horton ES Lebovitz H Levy P Moghissi ES Schwartz SS Statement by an American Association of Clinical Endocrinologists/American College of Endocrinology consensus panel on type 2 diabetes mellitus: an algorithm for glycemic control Endocr Pract 2009 15 540 559 19858063 10.4158/EP.15.6.540

[B5] Nathan DM Buse JB Davidson MB Ferrannini E Holman RR Sherwin R Zinman B Medical management of hyperglycemia in type 2 diabetes mellitus: a consensus algorithm for the initiation and adjustment of therapy: a consensus statement from the American Diabetes Association and the European Association for the Study of Diabetes Diabetologia 2009 52 17 30 10.1007/s00125-008-1157-y 18941734

[B6] Warsy AS el-Hazmi MA Diabetes mellitus, hypertension and obesity: common multi-factorial disorders in Saudis East Mediterr Health J 1999 5 1236 1242 11924118

[B7] Al-Nozha MM Al-Maatouq MA Al-Mazrou YY Al-Harthi SS Arafah MR Khalil MZ Khan NB Al-Khadra A Al-Marzouki K Nouh MS Abdullah M Attas O Al-Shahid MS Al-Mobeireek A Diabetes mellitus in Saudi Arabia Saudi Med J 2004 25 1603 1610 15573186

[B8] Yamaoka K Tango T Efficacy of lifestyle education to prevent type 2 diabetes Diabetes Care 2005 28 2780 2786 10.2337/diacare.28.11.2780 16249558

[B9] Alzaid A Time to declare war on diabetes Ann Saudi Med 1997 17 154 155 17377420 10.5144/0256-4947.1997.154

[B10] Fatani HH Mira SA El-Zubier AG Prevalence of diabetes mellitus in rural Saudi Arabia Diabetes Care 1987 10 180 183 10.2337/diacare.10.2.180 3582078

[B11] El-Hazmi M Warsy A Al-Swailem A Sulaimani R Diabetes mellitus as a health problem in Saudi Arabia East Mediterr Health J 1998 4 58 67

[B12] Gilles CL Abrams KR Lambert PC Cooper NJ Sutton AJ Hsu RT Khunti K Pharmacological and lifestyle interventions to prevent or delay type 2 diabetes in people with impaired glucose tolerance: systematic review and meta-analysis BMJ 2007 334 299 10.1136/bmj.39063.689375.55 17237299 PMC1796695

[B13] Wilson GA Gyi AA The status and perspective of diabetes health education in China: inspiration from Australia Int J Nurs Pract 2010 16 92 98 10.1111/j.1440-172X.2010.01817.x 20487053

[B14] Al-Nozha MM Al-Mazrou YY Al-Maatouq MA Arafah MR Khalil MZ Khan NB Al-Marzouki K Abdullah MA Al-Khadra AH Al-Harthi SS Al-Shahid MS Al-Mobeireek A Nouh MS Obesity in Saudi Arabia Saudi Med J 2005 26 824 829 15951877

[B15] Al-Nozha MM Arafah MR Al-Mazrou YY Al-Maatouq MA Khan NB Khalil MZ Al-Khadra AH Al-Marzouki K Abdullah MA Al-Harthi SS Al-Shahid MS Nouh MS Al-Mobeireek A Coronary artery disease in Saudi Arabia Saudi Med J 2004 25 1165 1171 15448760

[B16] Al-Nozha MM Abdullah M Arafah MR Khalil MZ Khan NB Al-Mazrou YY Al-Maatouq MA Al-Marzouki K Al-Khadra A Nouh MS Al-Harthi SS Al-Shahid MS Al-Mobeireek A Hypertension in Saudi Arabia Saudi Med J 2007 28 77 84 17206295

[B17] Chobanian AV Bakris GL Black HR Cushman WC Green LA Izzo JL Jr Jones DW Materson BJ Oparil S Wright JT Jr Roccella EJ Joint National Committee on Prevention, Detection, Evaluation and Treatment of High Blood Pressure. National Heart, Lung, and Blood Institute; National High Blood Pressure Education Program Coordinating Committee Seventh report of the Joint National Committee on Prevention, Detection, Evaluation, and Treatment of High Blood Pressure Hypertension 2003 42 1206 1252 10.1161/01.HYP.0000107251.49515.c2 14656957

[B18] Cole TJ Bellizzi MC Flegal KM Dietz WH Establishing a standard definition for child overweight and obesity worldwide: international survey BMJ 2000 320 1240 1246 10.1136/bmj.320.7244.1240 10797032 PMC27365

[B19] Falkner B Daniels SR Summary of the Fourth Report on the Diagnosis, Evaluation, and Treatment of High Blood Pressure in Children and Adolescents Hypertension 2004 44 387 388 10.1161/01.HYP.0000143545.54637.af 15353515

[B20] Shaw JE Sicree RA Zimmet PZ Global estimates of the prevalence of diabetes for 2010 and 2030 Diabetes Res Clin Pract 2010 87 4 14 10.1016/j.diabres.2009.10.007 19896746

[B21] Al-Daghri NM Al-Attas OS Alokail MS Alkharfy KM Sabico SL Chrousos GP Decreasing prevalence of the full metabolic syndrome but a persistently high prevalence of dyslipidemia among adult Arabs PLoS One 2010 5 e12159 10.1371/journal.pone.0012159 20730053 PMC2921394

[B22] Al-Daghri NM Extremely high prevalence of metabolic syndrome manifestations among Arab youth: a call for early intervention Eur J Clin Invest 2010 40 1063 1066 10.1111/j.1365-2362.2010.02341.x 20624169

[B23] Elsammak MY Al-Wosaibi AA Al-Howeish A Alsaeed J Vitamin D deficiency in Saudi Arabs Horm Metab Res 2010 42 364 368 10.1055/s-0030-1248296 20213587

[B24] Al-Daghri NM Al-Attas OS Alokail MS Alkharfy KM Al-Yousef MA Nadhrah HM Sabico SB Chrousos GP Severe hypovitaminosis D is widespread and more common in non-diabetics than diabetics in Saudi adults Saudi Med J 2010 31 775 780 20635011

[B25] Al-Daghri NM Acute post cessation smoking: a strong predictive risk factor for metabolic syndrome among adult Saudis Saudi Med J 2009 30 267 271 19198718

[B26] Bawazeer NM Al-Daghri NM Valsamakis G Al-Rubeaan KSA Sabico SL Kumar S McTernan PG Harte AL Sleep duration and quality associated with obesity among Arab children Obesity (Silver Spring) 2009 17 2251 2253 10.1038/oby.2009.169 19498352

[B27] Al-Hussein FA Diabetes control in a primary care setting: a retrospective study of 651 patients Ann Saudi Med 2008 28 267 271 10.4103/0256-4947.51700 18596404 PMC6074353

[B28] Aljoudi AS Taha AZ Knowledge of diabetes risk factors and preventive measures among attendees of a primary care center in eastern Saudi Arabia Ann Saudi Med 2009 29 15 19 10.4103/0256-4947.51813 19139622 PMC2813608

[B29] Al-Nozha MM Al-Hazzaa HM Arafah MR Al-Khadra A Al-Mazrou YY Al-Maatouq MA Khan NB Al-Marzouki K AL-Harthi SS Abdullah M Al-Shahid MS Prevalence of physical activity and inactivity among Saudis aged 30-70 years: a population-based cross-sectional study Saudi Med J 2007 28 559 568 17457478

[B30] Al-Hamdan N Saeed A Kutbi A Choudhury AJ Nooh R Characteristics, risk factors, and treatment practices of known adult hypertensive patients in Saudi Arabia Int J Hypertens 2011 2010 168739 21318133 10.4061/2010/168739 PMC3034950

[B31] Elhadd TA Al-Amoudi AA Alzahrani AS Epidemiology, clinical and complications profile of diabetes in Saudi Arabia: a review Ann Saudi Med 2007 27 241 250 10.4103/0256-4947.51484 17684435 PMC6074292

[B32] Chrousos GP The role of stress and the hypothalamic-pituitary-adrenal axis in the pathogenesis of the metabolic syndrome: neuroendocrine and target-tissue-related causes Int J Obes Relat Metab Disord 2000 24 S50 S55 10997609 10.1038/sj.ijo.0801278

[B33] El-Hazmi MA Al-Swailem A Warsy AS Al-Sudairy F Sulaimani R Al-Swailem A Al-Meshari A The prevalence of diabetes mellitus and impaired glucose tolerance in the population of Riyadh Ann Saudi Med 1995 15 598 601 17589018 10.5144/0256-4947.1995.598

[B34] Bassiony MM Smoking in Saudi Arabia Saudi Med J 2009 30 876 881 19617999

[B35] Al-Rethaiaa AS Fahmy AE Al-Shwaiyat NM Obesity and eating habits among college students in Saudi Arabia: a cross-sectional study Nutr J 2010 9 39 10.1186/1475-2891-9-39 20849655 PMC2949783

[B36] Al-Daghri NM Al-Attas OS Alokail MS Alkharfy KM Yousef M Sabico S Chrousos GP Hypovitaminosis D and cardiometabolic risk factors among non-obese youth Cent Eur J Med 2010 5 752 757 10.2478/s11536-010-0045-2

[B37] Al-Attas OS Al-Daghri NM Alokail MS Al-Fadda A Bamakhramah A Sabico S Pritlove D Harte A Tripathi G McTernan PG Kumar S Chrousos GP Adiposity and insulin resistance correlate with telomere length in middle-aged Arabs: the influence of circulating adiponectin Eur J Endocrinol 2010 163 601 607 10.1530/EJE-10-0241 20679357 PMC2938925

[B38] Al-Nuaim AR Prevalence of glucose intolerance in urban and rural communities in Saudi Arabia Diabet Med 1997 17 293 297 10.1002/(SICI)1096-9136(199707)14:7<595::AID-DIA377>3.0.CO;2-C 9223399

[B39] Al-Daghri NM Al-Attas OS Alokail MS Alkharfy KM Yakout SM Sabico S Gibson G Chrousos GP Kumar S Parent-offspring transmission of adipocytokine levels and their associations with metabolic traits PLoS One 2011 6 e18182 10.1371/journal.pone.0018182 21483749 PMC3070726

[B40] Al-Rowailly MA Abolfotouh MA Predictors of gestational diabetes mellitus in a high-parity community in Saudi Arabia East Mediterr Health J 2010 16 636 641 20799591

